# Fault Detection Using the Clustering-kNN Rule for Gas Sensor Arrays

**DOI:** 10.3390/s16122069

**Published:** 2016-12-06

**Authors:** Jingli Yang, Zhen Sun, Yinsheng Chen

**Affiliations:** 1 School of Electrical Engineering and Automation, Harbin Institute of Technology, Harbin 150080, China; hitsunz@163.com (Z.S.); chen_yinsheng@126.com (Y.C.); 2 Department of Electrical and Computer Engineering, McGill University, Montreal, QC H3A 0E9, Canada

**Keywords:** gas sensor arrays, fault detection, landmark-based spectral clustering, k-nearest neighbour rule

## Abstract

The k-nearest neighbour (kNN) rule, which naturally handles the possible non-linearity of data, is introduced to solve the fault detection problem of gas sensor arrays. In traditional fault detection methods based on the kNN rule, the detection process of each new test sample involves all samples in the entire training sample set. Therefore, these methods can be computation intensive in monitoring processes with a large volume of variables and training samples and may be impossible for real-time monitoring. To address this problem, a novel clustering-kNN rule is presented. The landmark-based spectral clustering (LSC) algorithm, which has low computational complexity, is employed to divide the entire training sample set into several clusters. Further, the kNN rule is only conducted in the cluster that is nearest to the test sample; thus, the efficiency of the fault detection methods can be enhanced by reducing the number of training samples involved in the detection process of each test sample. The performance of the proposed clustering-kNN rule is fully verified in numerical simulations with both linear and non-linear models and a real gas sensor array experimental system with different kinds of faults. The results of simulations and experiments demonstrate that the clustering-kNN rule can greatly enhance both the accuracy and efficiency of fault detection methods and provide an excellent solution to reliable and real-time monitoring of gas sensor arrays.

## 1. Introduction

The first application of gas sensor arrays was proposed by Persaud et al. [1] to discriminate between simple odours. Since then, countless efforts have been made to address the detection of gases with sensor arrays in many fields. The results to date indicate that semiconductor metal oxide (SMO) gas sensor arrays can provide a specific and unique response solution for different individual chemical gases or mixtures of gases [2,3]. Nowadays, gas sensor arrays are used in more and more engineering applications, such as chemical engineering, aerospace engineering and environmental engineering [4,5,6].

To maintain the concentrations of dangerous gases within the limits specified by regulations, the ability of reliable and real-time monitoring is extremely essential for gas sensor arrays. However, SMO gas sensors are prone to failures because of the nature of chemical sensors (sensing material degradation as a result of irreversible chemical reactions), including sensing layer ageing or poisoning or external effects (power supply instability or electrical interference) [7,8]. Moreover, the gas sensor array always faces a greater risk of failures because of the integrated redundant SMO gas sensors. Once a fault occurs, the faulty sensor should be detected in a timely manner. Therefore, many achievements on fault detection have been made to deal with failures of gas sensor arrays [9,10].

**Figure 1:**
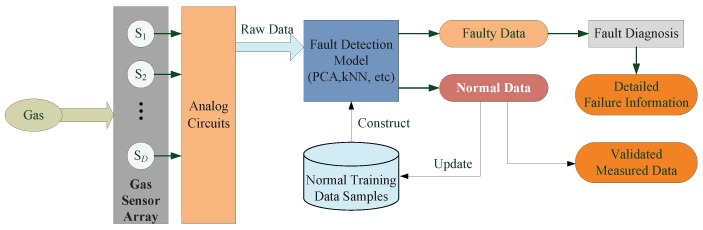
The framework of a data-driven fault detection method for gas sensor arrays.

Fault detection methods can be classified into two types: model-based and data-driven [11,12]. Model-based methods have to construct a model of the system to describe the real process, so it can only be efficient in less than 10% of real-world applications [13]. Different from model-based methods, data-driven methods are getting more and more attention because they only depend on the measured data samples [14]. The framework of a data-driven fault detection method for gas sensor arrays is shown in Figure 1. Normal (fault-free) data samples of gas sensor arrays are collected to build the fault detection model. The established model is disturbed if the statistics exceed their control limits; thus, at least one sensor of the system works in faulty mode. To locate faulty sensors, a fault diagnosis process can also be triggered.

Among existing data-driven methods, multivariate statistical techniques, such as principal component analysis (PCA), non-negative matrix factorization (NMF) and independent component analysis (ICA), have drawn increasing interest for the monitoring of output signals of gas sensor arrays [15,16,17]. The PCA method divides the original observation space into a principle component subspace (PCS) and a residual subspace (RS). On the arrival of a new test sample, it is first projected onto the PCS and RS, respectively. Then, Hotelling’s $T^{2}$ and the squared prediction error (SPE, also named *Q*) statistics are calculated, respectively [15]. If one of these statistics exceeds its control limit, the test sample is considered as faulty. Shen et al. [18] and Chen et al. [19] utilized the PCA method to solve fault detection problems of multifunctional sensors and gas sensor arrays, respectively.

It is known that the PCA method assumes that data samples are obtained from processes with linearity and follow a Gaussian distribution. However, it is difficult to satisfy these assumptions in practice. Furthermore, many chemical processes always have the features of non-linearity and multi-modality. To overcome the demerits of the PCA method, some improved methods for non-linear processes, such as the kernel principal component analysis (KPCA) [20] method, have been developed. The key idea of the KPCA method is to map the observation space into a high dimensional feature space via a non-linear mapping and conduct linear PCA in the feature space. Compared with PCA, KPCA is more flexible in extracting non-linear features, but it also leads to the degradation of the efficiency because of the complex operations in the high dimensional feature space [21].

Recently, the k-nearest neighbour (kNN) [22] rule has been applied successfully in fault detection. The kNN rule is one of the sample-based learning algorithms [23,24], whose purpose is to classify objects using nearest samples. Therein, the value of *k* indicates how many nearest neighbours are used for classification. He et al. [25] proposed the first kNN-based fault detection method named FD-kNN, and the idea behind FD-kNN is that a normal sample is similar to training samples collected in normal mode, while a fault sample deviates from training samples significantly. FD-kNN has no restriction for the distribution of data samples and can deal with the non-linearity and multi-modality of data [26]. To reduce the consumption of detection time and storage space, He et al. [27] presented PC-kNN, which is a principal component-based kNN method. PC-kNN firstly maps the original data into the principal component subspace; then, the kNN rule is applied to the score matrix to construct the fault detection model. Therefore, the consumption of time and storage space can be reduced greatly. However, the distances of samples in the original observation space cannot be maintained in the principle component subspace because the idea of using PCA is to express data samples using as few principle components as possible. Zhou et al. [28] proposed a fault detection method named RPkNN, which integrates the kNN rule and random projection to address the problems of multi-modality and non-linearity. The random projection can maintain the distances of data samples in the random subspace with great probability. Recently, Guo et al. [29] proposed a process-monitoring method named FS-kNN. This method projects the data samples into the feature space, where the principal components (*T*) and squared prediction error (SPE) are extracted as feature indicators. Feature indicators can effectively express the information of the raw data; thus, the detection accuracy of FS-kNN is higher than that of PC-kNN. For the calculation of the cumulative distances, all of the above methods simply use the Euclidean distance. However, this distance is not adaptive for each case because it neglects the relationships among variables. To resolve this problem, Verdier et al. [30] presented the k-NND method, which adopts an adaptive Mahalanobis distance for the calculation of the cumulative distances. The local covariance of data samples is adopted in the k-NND method for enhancing the adaptability of the kNN rule. The experimental results show that the Mahalanobis distance is more effective than the Euclidean distance.

Generally speaking, all of the above kNN-based fault detection methods (e.g., FD-kNN [25], PC-kNN [27], RPkNN [28], FS-kNN [29], k-NND [30]) select the k-nearest training samples as neighbours of the new test sample and calculate the average squared distance between the test sample and these neighbours. Therefore, the kNN rule has to calculate the distance between each training sample and the test sample in order to choose the k-nearest neighbours. The enormous time consumption prevents the above fault detection methods from being used in real-time monitoring applications [31]. To address this problem, this paper proposes a clustering-kNN rule to accomplish reliable and real-time fault detection of gas sensor arrays. The innovative contributions of this paper are summarized as follows.

(1) The k-nearest neighbour rule is introduced to solve the fault detection problem of gas sensor arrays for the first time. Based on this, the reliable detection of slight faults can be achieved because kNN naturally handles the possible non-linearity of the data samples.
(2) The clustering-kNN rule is proposed to improve the efficiency of the kNN rule by reducing the number of training samples involved in the detection process of each test sample. The landmark-based spectral clustering (LSC) algorithm is employed to divide the entire training sample set into several clusters, and the kNN rule is only performed in the cluster that is nearest to the test sample.
(3) The clustering-kNN rule is proposed to improve the efficiency of the kNN rule by reducing the number of training samples involved in the detection process of each test sample. The landmark-based spectral clustering (LSC) algorithm is employed to divide the entire training sample set into several clusters, and the kNN rule is only performed in the cluster that is nearest to the test sample.
(4) A real experimental system for gas sensor arrays is realized to perform a series of experiments, and the results show that the proposed clustering-kNN rule can greatly enhance both the accuracy and efficiency of fault detection methods.

This paper is organized as follows. Section 2 presents the details of the proposed clustering-kNN rule for efficient fault detection. In Section 3, simulations are carried out to verify the effectiveness of the proposed clustering-kNN rule and to analyse the effects of the parameters on the performance of fault detection methods. Section 4 demonstrates the performance of the proposed clustering-kNN rule in practice through an experimental system. Finally, the conclusion is given in Section 5.

## 2. Methodology

Admittedly, most existing kNN-based fault detection methods (e.g., FD-kNN [25], PC-kNN [27], FS-kNN [29] and k-NND [30]) are good at dealing with the non-linearity and multi-modality of data samples. However, these methods are time consuming because the kNN rule has to compute the distance between the test sample and each training sample. Therefore, they are not applicable for real-time monitoring applications, especially when there are a large number of training samples. To resolve this problem, the clustering-kNN rule is developed as follows.

### 2.1. Overview

The basic concept behind the clustering-kNN rule is to reduce the number of training samples involved in the detection process of each test sample by using the *k*-means clustering method. Similar to existing data-driven methods, the fault detection method based on the proposed clustering-kNN rule (referred to as clustering-kNN-based) is also made up of two processes, namely off-line model construction and on-line fault detection. The model construction process is designed to divide the entire training sample set into several clusters and to calculate the threshold for the fault detection statistic. The fault detection process is utilized to find the closest cluster for the test sample and to use all samples in this cluster as its calculation subset. Then, the fault detection statistic is achieved using the kNN rule on this new calculation subset. Considering that the calculation subset used for the kNN rule is just a small part of the entire training sample set, thus the efficiency of clustering-kNN-based fault detection methods can be enhanced.

Because the proposed clustering-kNN rule has the same function as the kNN rule, which means it can enhance the efficiency of all existing kNN-based fault detection methods, including FD-kNN, PC-kNN, FS-kNN and k-NND, to distinguish from existing methods, we name the corresponding clustering-kNN-based fault detection methods as C-FD-kNN, C-PC-kNN, C-FS-kNN and C-k-NND, respectively. Due to the limitation of space, we take the C-FD-kNN method as the example for illustrating the details of the proposed clustering-kNN rule. Without loss of generality, all other methods can be extended simply using the same principle.

### 2.2. Off-Line Model Construction

#### 2.2.1. The Landmark-Based Spectral Clustering

The clustering strategy is one of the basic methods in data mining, and it is proposed to classify samples into several clusters according to some common features. Specifically, samples stored in the same cluster are similar to each other, while samples stored in different clusters are dissimilar from each other. Although many clustering strategies have been developed for classification, they are still limited for real-time applications because of their high computational complexities [32].

Recently, Chen et al. [33] proposed the landmark-based spectral clustering (LSC) algorithm, which has low computational complexity. The basic concept of LSC is to choose a few data samples as landmarks and express other data samples using these landmarks. Compared with regular clustering algorithms that express each data sample with the entire data sample set, LSC can significantly save time consumption spent on achieving the affinity matrix. Moreover, LSC also has the merit that the computational complexity scales linearly.

The LSC algorithm compresses the entire data sample set by searching a series of typical elements and constructs the representation for each data sample, i.e., hunting for *P* representative samples. During the initial process, the random sampling or *k*-means method can be utilized to calculate the landmarks. The random sampling method randomly chooses the landmarks from the entire data sample set. The *k*-means method implements clustering on the entire data sample set and adopts the cluster centres as the landmarks.

In the beginning, each sample is adopted as the typical element to build up the landmark matrix *Z*. Suppose that $X=\left[x_{1},x_{2},...,x_{N}\right]\in {ℜ}^{S\times N}$ is a matrix that denotes the training samples of a gas sensor array, where *N* and *S* are the number of training samples and sensors, respectively. Furthermore, $x_{i}={\left[x_{1,i},x_{2,i},...,x_{S,i}\right]}^{T}\in {ℜ}^{S\times 1}$ is a vector that stands for the *i* training sample. LSC uses *P* landmarks to represent each data sample in the training sample set. Thus, we have to build a matrix *W* as the projection matrix of *X*, and the element of *W* is calculated as [33]:

(1) $$w_{ji}=\frac{K_{h}\left(x_{i},z_{j}\right)}{\sum_{l\in Z_{<i>}}K_{h}\left(x_{i},z_{l}\right)}\;\;,\;\;\;j\in Z_{<i>}$$

where $z_{j}$ is the *j* column vector of *Z* and $Z_{<i>}$ denotes a sub-matrix of *Z* that consists of *r* (r<P) nearest landmarks of $x_{i}$. $K_{h}\left(•\right)$ is the kernel function, and *h* is its bandwidth. The Gaussian kernel is a widely-used function, which can be expressed as follows.

(2) $$K_{h}\left(x_{i},z_{j}\right)=\exp\left(-{∥x_{i}-z_{j}∥}^{2}/2h^{2}\right)$$

Then, the spectral analysis is carried out, and the graph matrix *G* is achieved as follows.

(3) $$G={\hat{W}}^{T}\hat{W}$$

Here, matrix *G* can be eigen-decomposed efficiently. $\hat{W}=D^{-0.5}W$, where *D* is the row sum of *W*. It should be noted that the sum of each column of *W* is equal to one, and the degree matrix of *G* is the identify matrix.

Suppose $\hat{W}$ can be decomposed using the singular value decomposition (SVD) as follows.

(4) $$\hat{W}=U\Sigma V^{T}$$

where $\Sigma =diag\left(\sigma_{1},\sigma_{2},...,\sigma_{P}\right)$ and $\sigma_{1}\geq \sigma_{2}\geq ...\geq \sigma_{P}\geq 0$ are the singular values of $\hat{W}$, $U=\left[u_{1},u_{2},...,u_{P}\right]\in {ℜ}^{P\times P}$ are the left singular vectors and $V=\left[v_{1},v_{2},...,v_{P}\right]\in {ℜ}^{N\times P}$ are the right singular vectors.

Then, *U* can be achieved with a computational complexity of $O\left(P^{3}\right)$, and *V* can be achieved as follows.

(5) $$V^{T}=\Sigma^{-1}U^{T}\hat{W}$$

The computational complexity of *V* is $O\left(P^{3}+P^{2}N\right)$, which is significantly lower than $O\left(N^{3}\right)$ when P≪N. Each row of *V* is a sample, and all clusters can be achieved using the *k*-means strategy. Considering that the computational complexity declines from $O\left(N^{3}\right)$ to $O\left(N\right)$, thus the LSC algorithm is suitable to be used in real-time applications.

The pseudocode of the landmark-based spectral clustering algorithm is described as Algorithm 1.
**Algorithm 1** Landmark-based Spectral Clustering**Input**: Training samples $X=\left[x_{1},x_{2},...,x_{N}\right]$, clustering number *M* **Output**: *M* clusters, clusters centers $CP_{1}$,$CP_{2}$,...,$CP_{M}$ 1: Produce *P* landmark samples using random selection or *k*-means methods; 2: Construct a sparse affinity matrix *Z* between training data samples and landmarks, with the affinity matrix calculated based on Equation (1); 3: Calculate the first *M* eigenvectors of $WW^{T}$, denoted by $U=\left[u_{1},u_{2},...,u_{M}\right]$; 4: Calculate $V=\left[v_{1},v_{2},...,v_{M}\right]$ based on Equation (5); 5: Each row of *V* is a sample and *k*-means is adopted to achieve the clusters and cluster centers.

#### 2.2.2. The Statistic of the Clustering-kNN-Based Fault Detection Model

Once all samples in the entire training sample set have been classified into several clusters using the above LSC algorithm, the next task is to calculate the statistics for fault detection. Suppose that all training samples are stored in *M* clusters, and the number of training samples in the *m* (1≤m≤M) cluster is $C_{m}$. For the *i* training sample $x_{i}$ in the *m* cluster, the kNN rule is performed in this cluster:

(6) $$d_{i,j}^{2}={∥x_{i}-x_{j}∥}_{2}^{2},j=1,2,...,C_{m};j\neq i$$

where $d_{i,j}^{2}$ denotes the squared Euclidean distance between the *i* training sample and its *j* neighbour.

Therefore, the average squared distance between each sample and its neighbours is calculated as follows.

(7) $$D_{i}^{2}=\frac{1}{k}\sum_{j=1}^{k}d_{i,j}^{2}$$

where $D_{i}^{2}$ is called the kNN distance of $x_{i}$. The test sample is close to normal samples in the training sample set if all sensors work in normal mode; whereas the fault sample would present a certain distance from normal samples. Thus, the value of $D^{2}$ of a fault sample would be larger than that of a normal sample. Hence, $D^{2}$ has the potential to be used as the statistic for fault detection. It should also be noted that the calculation of the $D^{2}$ statistic in the clustering-kNN rule only involves training samples in the same cluster, which is also the main difference with the kNN rule.

If all data samples of a sensor array are independently identically distributed and obey the multivariate normal distribution, it is obvious that the distance between two samples would obey the chi-square distribution. However, this is impossible in practical applications. Considering that case, the threshold of the $D^{2}$ statistic cannot be determined directly from some classical distributions [25].

#### 2.2.3. The Threshold of the $D^{2}$ Statistic

As mentioned above, it is impossible to achieve the threshold of the $D^{2}$ statistic using a rigorous statistical distribution. Therefore, an approach named kernel density estimation (KDE) [34] is adopted to estimate the threshold of the $D^{2}$ statistic.

KDE is a powerful tool for estimating the probability density function (PDF), particularly for univariate random processes, such as the $D^{2}$ statistic in this work. Another merit of KDE is that the confidence region obtained using KDE follows the data more closely and is less likely to incorporate regions of unknown operation. Let $Y=\left\{y_{i}\right\}$, i=1,2,...,n denote a sample set, and its density function is $p\left(y\right)$. The latter can be expressed as follows.

(8) $$P\left(y<\alpha \right)=\int_{-\infty }^{\alpha }p\left(y\right)dy$$

In that case, the threshold for a specific confidence bound can be determined if Equation (8) has a certain expression. In contrast, the realistic method is to estimate the PDF as follows.

(9) $$\hat{p}\left(y\right)=\frac{1}{nh}\sum_{i=1}^{n}K\left(\frac{y-y_{i}}{h}\right)$$

where *n* is the number of samples, *h* is the bandwidth and $K\left(•\right)$ is a kernel function that conforms to:

(10) $$\int_{-\infty }^{+\infty }K\left(y\right)dy=1,\;\;\;\;K\left(y\right)\geq 0$$

The selection of the kernel function has a negligible effect on the result of the KDE estimation. In general, the Gaussian kernel is the most common choice. However, the bandwidth *h* strongly affects the accuracy of the KDE estimation. If *h* is too small, the density estimator will be too rough, whereas if it is too large, the density estimator will be too flat. In this paper, the generalized cross entropy (GCE) method [34] is adopted to choose the bandwidth *h*. In brief, the bandwidth *h* should satisfy the Csiszar measure as follows.

(11) $$\Phi \left(g\to \hat{p}\right)=\frac{1}{2}\int \frac{g^{2}\left(y\right)}{\hat{p}}dy-\frac{1}{2}$$

(12) $$g\left(y\right)=\hat{p}\left(y\right)\sum_{j=0}^{n}\lambda_{j}K_{j}\left(y\right)$$

where *λ* is the Lagrange multiplier. Therefore, the optimal bandwidth can be achieved by:

(13) $$h_{opt}={\left(2n\sqrt{\pi }{∥g^{{}^{\prime \prime }}\left(y,h\right)∥}^{2}\right)}^{-0.4}$$

Details of the GCE method can be found in [34,35]. Using the KDE approach, we can achieve the threshold of the $D^{2}$ statistic.

### 2.3. On-Line Fault Detection

Once *M* clusters and their centres $CP_{1},CP_{2},...,CP_{M}$ have been calculated by the LSC algorithm, the nearest cluster centre for the new test sample can be found out, and the corresponding cluster will be used as the calculation subset for the new test sample. Then, the kNN rule is performed in this cluster. With this method, the proposed clustering-kNN rule still can achieve high fault detection accuracy. The pseudocode of the fault detection process is listed in Algorithm 2.
**Algorithm 2** Fault Detection based on the Clustering-kNN rule**Input**: *M* clusters, clusters centers $CP_{1}$,$CP_{2}$,...,$CP_{M}$, new test sample $x_{new}$, the threshold $D_{\alpha }^{2}$ of the $D^{2}$ statistic **Output**: Fault detection result 1: Calculate the distance between the new test sample $x_{new}$ and each cluster center, denoted by $D\left(x_{new},CP_{i}\right)$, i=1,2,...,M; 2: Computer the nearest cluster center CP to $x_{new}$ according to $CP=\arg\min\left\{D\left(x_{new},CP_{i}\right)\right\}$, i=1,2,...,M; 3: Adopt the cluster of CP as the calculation subset of the new test sample $x_{new}$; 4: Apply the kNN algorithm to the calculation subset of $x_{new}$, and achieve the value of the $D^{2}$ statistic; 5: If the value of $D^{2}$ statistic is larger than the threshold $D_{\alpha }^{2}$, output the faulty work condition; otherwise, output the normal work condition.

As shown in Algorithm 2, when the number of clusters *M* is large, the clustering-kNN rule can reduce the computational complexity of the kNN rule and improve the efficiency of fault detection. However, the fault detection accuracy may decline with the increase of the number of clusters *M* because the possibility of the misclassification of training samples. By contrast, if *M* is small, i.e., M=1, the clustering-kNN rule decreases to the kNN rule, which is not suitable for real-time applications. Therefore, the number of cluster *M* should be selected with a reasonable value to keep a balance between the accuracy and the efficiency of fault detection; more details about the selection of *M* will be illustrated in Section 3.

## 3. Simulations

In this section, two examples with linear and non-linear models are conducted to show the ability of the proposed clustering-kNN rule under different situations, and the performance comparisons with the PCA method [19] and existing kNN-based fault detection methods (e.g., FD-kNN [25], PC-kNN [27], FS-kNN [29] and k-NND [30]) are also performed. Furthermore, the effects of parameters (e.g., the number of neighbours *k*, the number of clusters *M*) on the performance of fault detection methods are thoroughly analysed.

### 3.1. Linear Model

The first example is a linear case with the process model that has been adopted in [26]:

(14) $$x=\left[\begin{array}{ccc} -0.3441 & 0.4815 & 0.6637\\ -0.2313 & -0.5936 & 0.3545\\ -0.5060 & 0.2495 & 0.0739\\ -0.5552 & -0.2405 & -0.1123\\ -0.3371 & 0.3822 & -0.6115\\ -0.3877 & -0.3868 & -0.2035 \end{array}\right]\left[\begin{array}{c} t_{1}\\ t_{2}\\ t_{3} \end{array}\right]+noise$$

where variables $t_{1}$, $t_{2}$ and $t_{3}$ are uniformly distributed in the range of $\left[0,2.0\right]$, $\left[0,1.6\right]$ and $\left[0,1.2\right]$, respectively. The noise obeys the normal distribution with zero mean and a standard deviation of 0.2, and the number of generated training samples is 2000.

The test samples consist of validation samples and fault samples. The validation samples are normal samples generated from the linear model and used to verify the false positive detection rate (FPR) of each method. For fault samples, the simulated form is defined as:

(15) $$x_{f}=x+\xi_{s}f_{s}$$

where the fault direction $\xi_{s}$ is randomly selected from the above six possible variables and the fault amplitude $f_{s}$ obeys the uniform distribution in the range $\left[0,5.0\right]$. The numbers of the generated validation samples and fault samples are 1000, respectively.

The PCA method [19] is implemented to demonstrate the applicability of the kNN-based and clustering-kNN-based fault detection methods. The number of principal components (PCs) of the PCA-based detection model is determined using cumulative percent variance (CPV) with a value of 80%, and the statistics used to monitor the process are *Q* and $T^{2}$ with a confidence level of 99%. In addition, training samples are also used to construct the models of the kNN-based fault detection methods (e.g., FD-kNN [25], PC-kNN [27] and FS-kNN [29], k-NND [30]) and the clustering-kNN-based fault detection methods (e.g., C-FD-kNN, C-PC-kNN, C-k-NND, C-FS-kNN), and the thresholds of their $D^{2}$ statistics are determined with a confidence level of 99%, respectively.

Please note that the number of neighbours *k* is the critical parameter of the kNN classification algorithm, and it also has a major effect on the performance of the kNN-based and clustering-kNN-based fault detection methods. In other words, a large value of *k* can increase the robustness against noises, whereas a small value of *k* can enhance the sensitivity to slight faults [23]. Generally speaking, the value of *k* should be smaller than the square root of the number of training samples [36]. Therefore, quantitative analysis for the effects of the number of neighbours on the performance of fault detection methods is done by changing the value of *k* in the range [2,$\sqrt{N}$] to get different simulation results, where N=2000 is the number of training samples.

**Figure 2:**
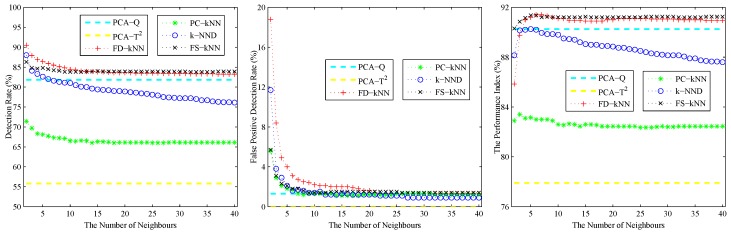
The effects of the number of neighbours of the linear model.

As shown in the left sub-graph of Figure 2, the detection rate (DR) of each kNN-based fault detection method declines with the increase of *k*. The reason for this phenomenon is caused by the effects of the average squared distance on a large number of neighbours. A similar phenomenon also occurs on the false positive detection rate (FPR), which is shown in the middle sub-graph of Figure 2. With the increase of the number of neighbours, the effects of noises on the detection results are also weakened. In conclusion, a larger *k* can help to reduce the false positive detection rate caused by noises; however, it also introduces the possibility of additional undetected errors, which means that slight faults would probably be neglected. By contrast, a smaller *k* can enhance the detection rate of slight faults, but it also enlarges the effects of noises on the detection results.

In order to maintain a balance between the robustness against noises and the sensitivity to faults, a cross-validation algorithm is adopted to determine the optimal value of *k*. Inspired by [11,37], a performance index Ω is defined as the evaluation criterion for the cross-validation algorithm.

(16) $$\Omega =\frac{\Upsilon \left(TP\right)+\Upsilon \left(TN\right)}{\Upsilon \left(TP\right)+\Upsilon \left(TN\right)+\Upsilon \left(FN\right)+\Upsilon \left(FP\right)}$$

where TP (normal samples detected as normal), TN (fault samples detected as faulty), FN (fault samples detected as normal) and FP (normal samples detected as faulty) are detection status, and $\Upsilon \left(S\right)$ is the number of samples in status *S*.

The performance index under different values of *k* is shown in the right sub-graph of Figure 2. As shown in this sub-graph, the performance index of each kNN-based fault detection method first increases then decreases with the number of neighbours. Therefore, the value at the maximum of the performance index is chosen as the optimal value of *k* for each fault detection method. In this model, the values of *k* for FD-kNN/C-FD-kNN, PC-kNN/C-PC-kNN, k-NND/C-k-NND and FS-kNN/C-FS-kNN are set to 6, 3, 5, 5, respectively.

In addition to the number of neighbours *k*, the number of clusters *M* is the other important parameter of the proposed clustering-kNN rule. On the one hand, the computational complexity of the clustering-kNN rule depends on the number of training samples in a cluster; thus, the efficiency of the clustering-kNN-based fault detection method increases with the increase of *M*. On the other hand, the clustering-kNN rule is a local search algorithm based on a cluster of the entire training sample set; thus, the accuracy of the clustering-kNN-based fault detection method declines with the increase of *M* because of the possibility of the misclassification of training samples. However, the clustering-kNN rule can achieve the same results with the kNN rule when the nearest *k* neighbours of a test sample are classified in the cluster that is nearest to it. Therefore, the number of clusters *M* depends on the similarity of training samples in the same cluster and the dissimilarity of training samples in different clusters. When samples in the same cluster are similar to each other and samples in different clusters are dissimilar from each other, a larger *M* can be adopted to enhance the efficiency of fault detection methods. Otherwise, a smaller *M* should be used to keep a balance between the efficiency and the accuracy of the fault detection methods.

**Figure 3:**
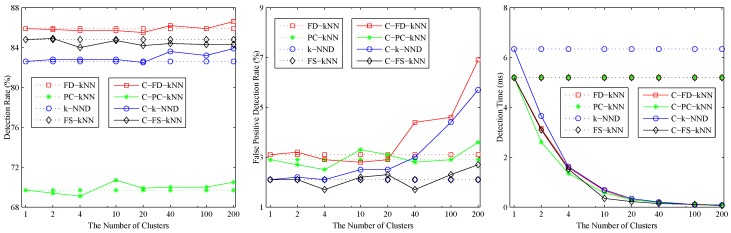
Effects of the number of clusters of the linear model.

The effects of the number of clusters *M* on clustering-kNN fault detection methods are shown in Figure 3. As shown, when M<20, the detection rates and false positive detection rates of C-FD-kNN, C-PC-kNN, C-k-NND, C-FS-kNN are almost the same as those of FD-kNN, PC-kNN, k-NND, FS-kNN, respectively. At the same time, the detection time of the clustering-kNN-based fault detection methods drops sharply with the increase of *M*, which means that the proposed clustering-kNN rule can enhance the efficiency of fault detection without reducing the accuracy. In this model, the value of *M* for each clustering-kNN-based fault detection method (e.g., C-FD-kNN, C-PC-kNN, C-k-NND, C-FS-kNN) is set to 20.

**Figure 4:**
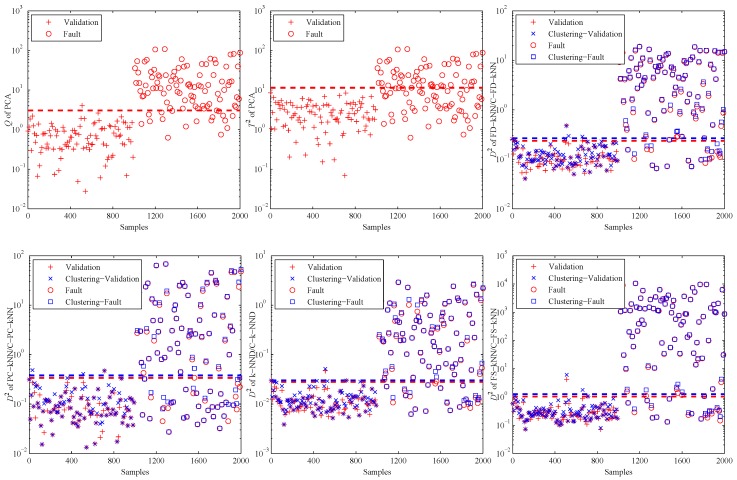
Fault detection results of the linear model.

Figure 4 shows the fault detection results of this linear model, and it is obvious that most methods have good performance on the detection of fault samples. The *Q* statistic of the PCA method performs similarly to FD-kNN, and the $T^{2}$ statistic is less effective because it is insensitive for slight faults. In addition, the $D^{2}$ statistic of each clustering-kNN-based fault detection method is similar to that of the corresponding kNN-based fault detection method, which means our proposed clustering-kNN rule has negligible influences on the detection accuracy.

As shown in Table 1, four metrics, including model construction time ($T_{C}$), detection time ($T_{D}$), detection rate (DR) and false positive detection rate (FPR), are employed to demonstrate more details about the detection results. Compared with the kNN-based fault detection methods, the clustering-kNN-based fault detection methods consume less time on model construction and detection. This improvement is due to the clustering-kNN rule using the LSC algorithm to reduce the number of training samples that are involved in the detection process of each test sample. Specifically, the detection speed of C-FD-kNN is about 18-times that of FD-kNN in this linear model.

### 3.2. Non-Linear Model

The second example is the non-linear case that has been adopted in [21]:

(17) $$\left\{\begin{array}{c} x_{1}=t+e_{1}\;\;\;\;\;\;\;\;\;\;\;\\ x_{2}=t^{2}-3t+e_{2}\;\;\;\\ x_{3}=-t^{3}+3t^{2}+e_{3} \end{array}\right.$$

where $e_{1}$, $e_{2}$ and $e_{3}$ are independent white noises with zero mean and a standard deviation of 0.01, and *t* is uniformly distributed in the range of $\left[0.01,2.0\right]$.

**Figure 5:**
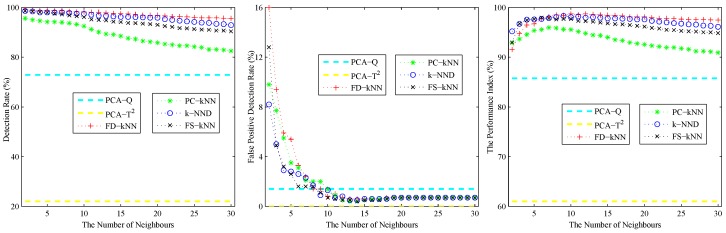
Effects of the number of neighbours of the non-linear model.

Similar to the linear case, 1000 samples are used for training; 1000 samples are used for validation; and 1000 fault samples introduced according to Equation (15) are used for detection. Figure 5 shows the effects of the number of neighbours of the non-linear model. As shown, the detection rate and false positive detection rate of each kNN-based fault detection method decline with the increase of *k*. The performance index of each kNN-based fault detection method first increases, then decreases with the the increase of *k*. According to the principle of maximizing the performance index, the values of *k* for FD-kNN/C-FD-kNN, PC-kNN/C-PC-kNN, k-NND/C-k-NND and FS-kNN/C-FS-kNN are set to 10, 7, 9, 6, respectively.

**Figure 6:**
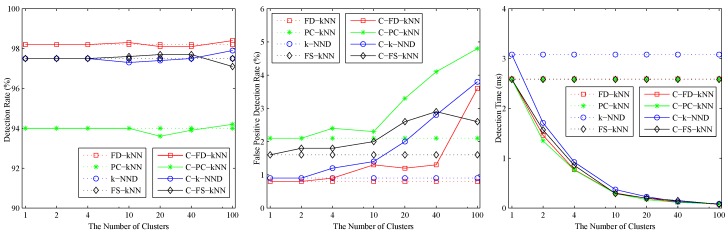
Effects of the number of clusters of the non-linear model.

The effects of the number of clusters on clustering-kNN-based fault detection methods are shown in Figure 6. When M<10, the detection rate and false positive detection rate of C-FD-kNN are in accordance with that of FD-kNN, and similar things also happen in the other pairs (e.g., C-PC-kNN and PC-kNN, C-k-NND and k-NND, C-FS-kNN and FS-kNN). In addition, the detection time of clustering-kNN-based fault detection methods is far less than that of kNN-based fault detection methods. In this model, the value of *M* for each clustering-kNN-based fault detection method is set to 10.

**Figure 7:**
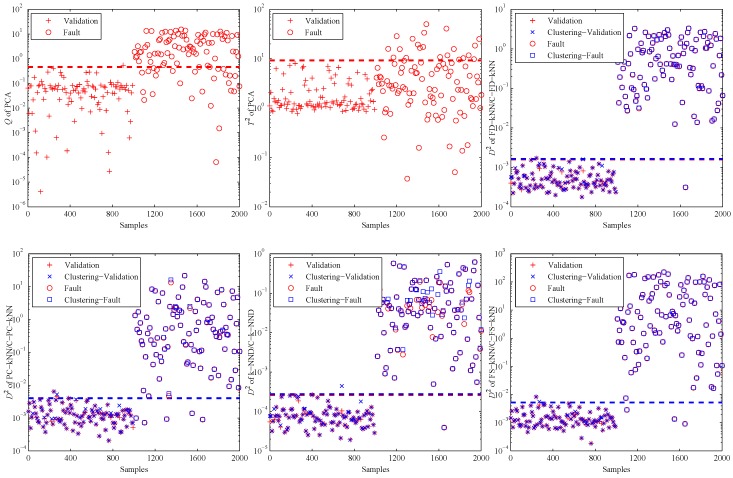
Fault detection results of the non-linear model.

When the PCA method is used to detect the validation and fault samples with a confidence level of 99%, the detection results are shown in the first two sub-graphs of Figure 7. It is obvious that most of the fault samples are not detected by PCA, especially for the $T^{2}$ statistic. That is due to the non-linearity of data samples. This example illustrates in which levels the non-linearity can affect the fault detection accuracy of PCA.

Then, the kNN-based and clustering-kNN-based fault detection methods are implemented on the same dataset, and the results are shown in the last four sub-graphs of Figure 7. It should be noted that these methods can reveal the non-linearity of data samples by choosing the nearest neighbours.

More details about the detection results of the non-linear model are shown in Table 2. Similar to the linear case, the proposed clustering-kNN-based fault detection methods also spend less time on model construction and detection. Specifically, the detection speed of the C-FD-kNN is about nine-times that of the FD-kNN in this non-linear model.

## 4. Experiments and Results

To validate the ability of the proposed clustering-kNN rule in practice, a real experimental system for gas sensor arrays is constructed. Fault detection methods based on the clustering-kNN rule are evaluated under both cases of different types of faults and different amplitudes of faults.

### 4.1. Experimental Setup

**Figure 8:**
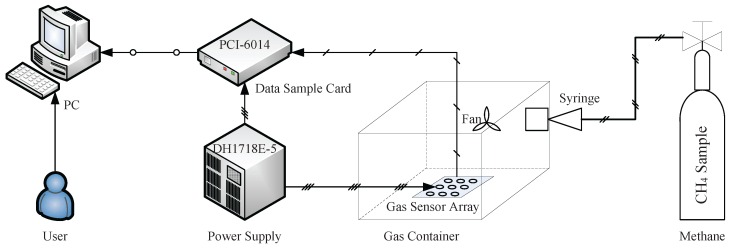
The experimental system for gas sensor arrays.

The structure of the experimental system is shown in Figure 8, which consists of a gas sensor array, a data sample card, a power device, a gas source of methane and a PC. The gas sensor array with sixteen sensors is placed into a gas container. CH${}_{4}$ is chosen as the gas sample, and it is poured into the container with a syringe. The gas samples are distributed uniformly throughout the gas container using a fan. The concentration of CH${}_{4}$ samples ranges from 50 ppm to 2000 ppm in our experiments. Measuring circuits are designed to convert the physical measurement parameters to analogue signals, and the data sample process is realized on a PC with the data sample card.

According to long-term observation and experience, the output signals of gas sensor arrays are of several types of faults: bias fault, impact fault, constant output fault and broken-circuit fault. When a constant output fault occurs, the faulty sensor has no response to the gas, and the output signals are similar to the response in clean air. In the process of the normal mode of gas sensor arrays, its power supply is removed to generate a broken-circuit fault, which is very common in practical applications because of a fault signal electrode and poor soldering. When an external disturbance occurs, the sensor with high resistance is affected and a short-term impact faults happens. Bias fault is used to simulate the effect from a long-term poorly measured environment. Considering that the signal data of a broken-circuit fault seem like a constant output fault with the amplitude near zero, they are treated as a special case of the constant output fault in our experiments [18,19].

### 4.2. Model Construction

The normal mode of gas sensor arrays is the condition that all sensors are fault free, and training samples used for constructing fault detection models are collected in this mode. In our experiments, training samples are collected with 40 different concentration levels that increase at a regular interval (50 ppm), and the sample time of each concentration level lasts for 200 s.

The parameters of all fault detection methods are described as follows: the number of principal components (PCs) of the PCA method is determined by cumulative percent variance (CPV) with a value of 90%, and the $T^{2}$ and *Q* statistics are used to monitor the output signals of the gas sensor array with a confidence level of 99%. The threshold of the $D^{2}$ statistic in each kNN-based or clustering-kNN-based fault detection method is calculated with a confidence level of 99%. The training samples collected in a sample concentration level have much in common, whereas that collected in different concentration levels shows a greater distinction. Therefore, the number of clusters *M* used in each clustering-kNN-based fault detection method (e.g., C-FD-kNN, C-PC-kNN, C-k-NND, C-FS-kNN) is set to 40, which can guarantee that it can achieve the same detection accuracy as the corresponding kNN-based fault detection method.

It should be noted that the kNN rule and the proposed clustering-kNN rule adopted in this paper are effective under the assumption that training samples are balanced, which means the number of training samples that are collected in each concentration level is the same. For the case of unbalanced training samples, the weighted kNN rule [38,39] can be adopted to address this problem.

**Figure 9:**
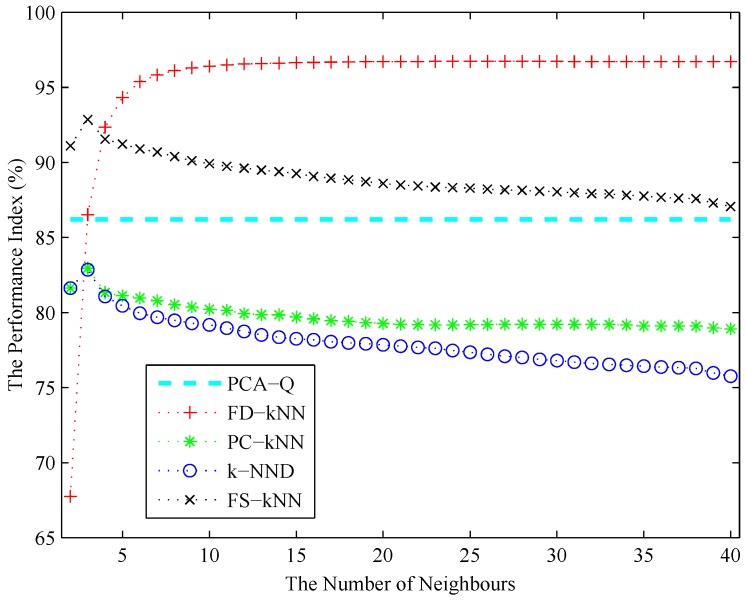
The performance index versus the number of neighbours.

The number of neighbours *k* of each kNN-based fault detection method (e.g., FD-kNN, PC-kNN, k-NND and FS-kNN) is determined using the cross-validation algorithm as described in Section 3. As shown in Figure 9, the performance index of each fault detection method increases initially and then decreases with the increase of *k*. According to the principle of maximizing the performance index, the values of *k* for FD-kNN, PC-kNN, k-NND and FS-kNN are set to 30, 3, 3, 3, respectively. To achieve the sample fault detection accuracy, each clustering-kNN-based fault detection method takes the same value of *k* as the corresponding kNN-based fault detection method; thus, the values of *k* for C-FD-kNN, C-PC-kNN, C-k-NND and C-FS-kNN are set to 30, 3, 3, 3, respectively.

### 4.3. Fault Detection

**Figure 10:**
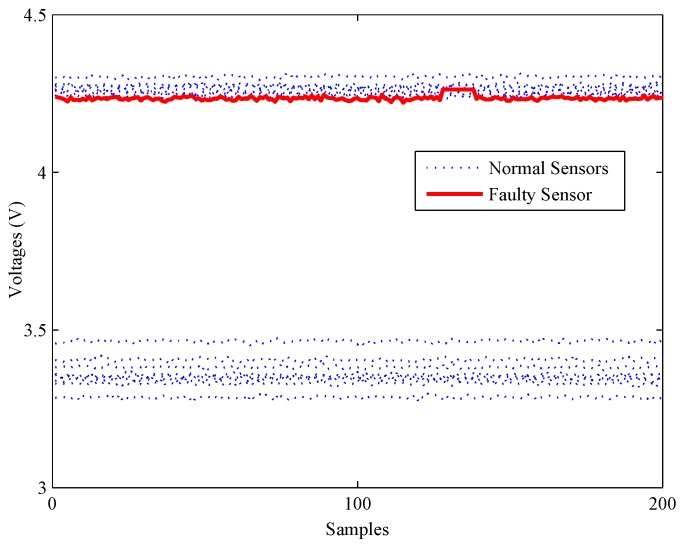
A slight impact fault of the gas sensor array.

**Figure 11:**
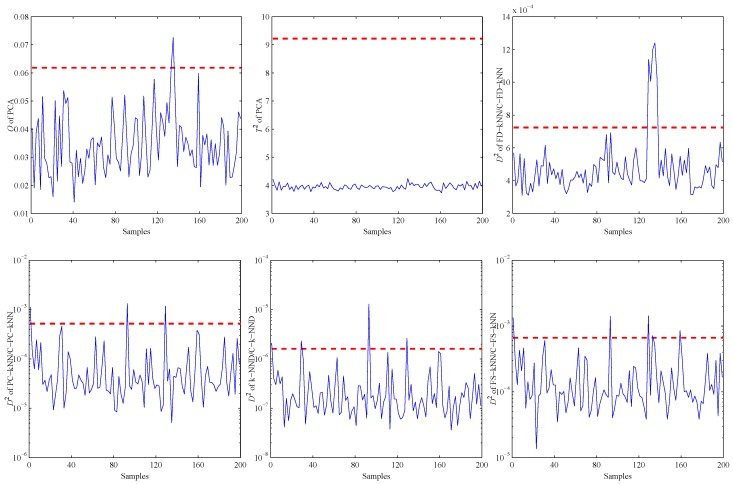
Detection results of the slight impact fault.

Firstly, an experiment is performed when the CH${}_{4}$ concentration is 200 ppm to validate the performance of all methods on detecting impact faults. As shown in Figure 10, a slight impact fault of the No. 9 sensor is simulated by adding eleven large data (about 0.5% of the mean value) at about 130 s. Figure 11 shows the detection results of this experiment. As shown, it is almost impossible for the PCA-based fault detection method to make a distinction between fault signals and normal signals by using the *Q* statistic. Furthermore, its $T^{2}$ statistic almost has no fluctuation during the entire process, which means no fault is detected by this statistic.

By contrast, the $D^{2}$ statistics of FD-kNN and C-FD-kNN show a sharp increase at the time the fault occurs, and all values also exceed their threshold, respectively. Therefore, this slight impact fault is detected by the FD-kNN and C-FD-kNN methods. It should be noted that training samples have high similarity on each concentration level and low similarity between different concentration levels. Therefore, the LSC algorithm introduces no misclassification of training samples; thus, C-FD-kNN achieves the same fault detection accuracy as FD-kNN.

**Figure 12:**
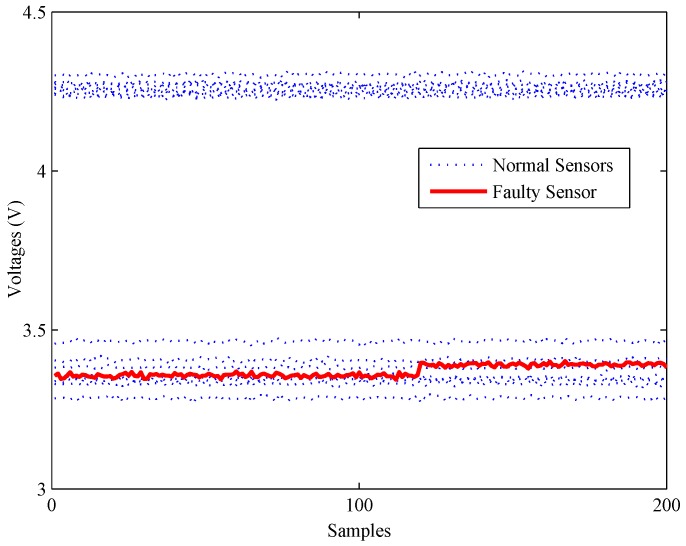
A slight bias fault of the gas sensor array.

**Figure 13:**
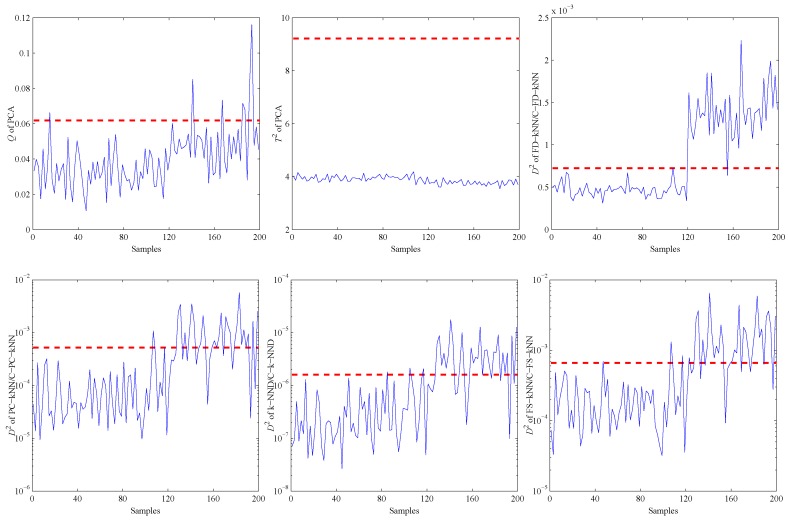
Detection results of the slight bias fault.

Secondly, another experiment is carried out to illustrate the detection ability of all methods under bias faults. As shown in Figure 12, a bias fault of the No. 13 sensor is simulated by adding a constant value (about 1% of the mean value) at about 120 s. The fault detection results of the bias fault are shown in Figure 13.

The $D^{2}$ statistics of the FD-kNN and C-FD-kNN methods are larger than their thresholds since the time the fault occurs, respectively, which means that these two methods can detect this bias fault. As for the PCA-based method, its *Q* statistic rises sharply at the time the fault occurs, but most of the values are still below its upper control limit. Therefore, most fault samples are neglected by the PCA-based method.

**Figure 14:**
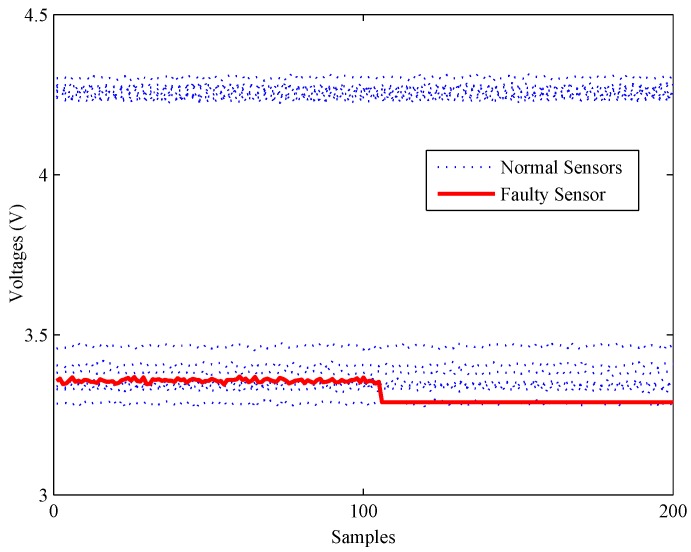
A slight constant output fault of the gas sensor array.

The third experiment on fault detection is conducted to verify the detection ability of all methods under constant output faults. As shown in Figure 14, a constant output fault of No. 16 is simulated by setting its output data to a constant value (about 98% of the mean value) at about 110 s.

**Figure 15:**
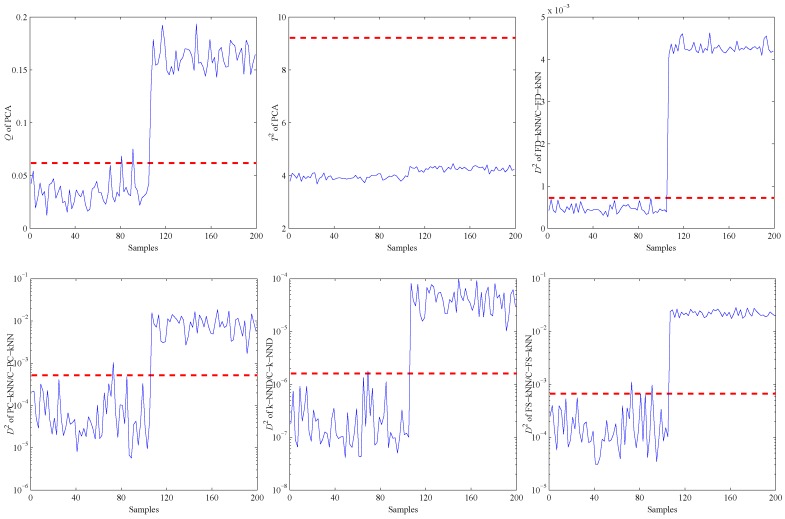
Detection results of the slight constant output fault.

The fault detection results of different methods under the constant output fault are shown in Figure 15. All statistics excluding the $T^{2}$ statistic of the PCA-based method exceed their thresholds or upper control limits, which means this fault is detected by all of these methods.

In conclusion, the detection result of each fault detection method is related to the amplitude of the fault. Intuitively, the possibility for a fault being detected increases with its amplitude. To illustrate to what degree each detection method is affected by the amplitude of the fault, a series of experiments is conducted under different CH${}_{4}$ concentration levels. During these experiments, the fault factor *θ*, which is defined as the ratio of *f* and *u*, ranges from 0.005 to 0.02, where *u* is the mean value of the normal signal of a sensor in a certain concentration level and *f* is the amplitude of the fault occurring in this sensor.

**Figure 16:**
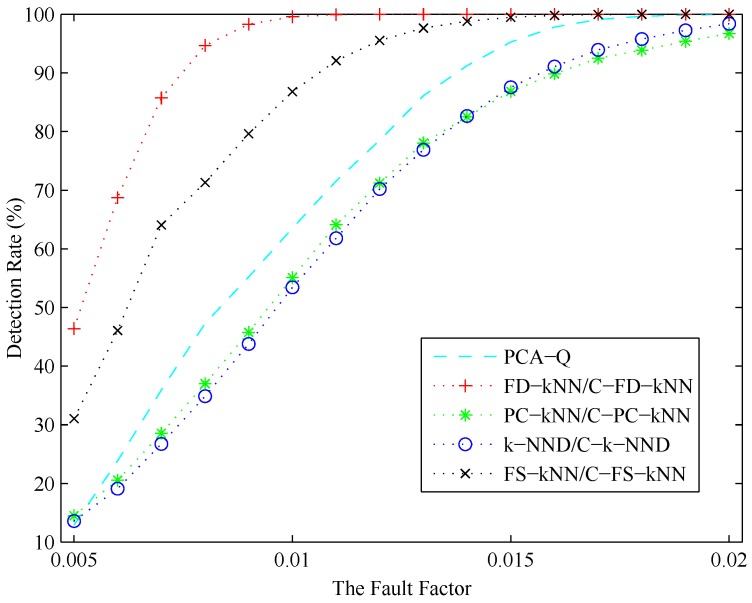
Detection rates versus the fault factors.

As shown in Figure 16, the detection rate (DR) of each fault detection method increases with the fault factor. A stable fault detection rate can be obtained by FS-kNN and C-FS-kNN when the fault factor is larger than 0.015, respectively, while that of the PCA method is about 0.018. When the fault factor of a fault is larger than 0.01, this fault can be detected by FD-kNN and C-FD-kNN definitely. Moreover, the FD-kNN and C-FD-kNN methods can still achieve relatively high detection rates even when the fault factor is less than 0.005, which means the kNN-based and clustering-kNN-based fault detection methods are more effective in detecting slight faults. Although the storage space consumption can be reduced by PC-kNN and C-PC-kNN, the distances of data samples in the original space cannot be maintained in the principle component subspace. That means the the detection rate of PC-kNN may be lower than that of FD-kNN, which conforms to the results in Figure 16. FS-kNN and C-FS-kNN have better performance than PC-kNN and C-PC-kNN on detection rates, which is due to the feature space containing much more information than the principle component space.

Table 3 shows the false positive detection rate (FPR) of each fault detection method. As shown, the FD-kNN and C-FD-kNN methods can achieve good performance on this metric, which is close to the PCA-based method. Therefore, the FD-kNN and C-FD-kNN methods can keep an excellent balance between the robustness against noises and the sensitivity to faults.

Compared with kNN-based fault detection methods, clustering-kNN-based fault detection methods consume less time on model construction and detection; thus, our proposed clustering-kNN rule can enhance the efficiency of the fault detection. As shown in Table 4, the detection speed of each clustering-kNN-based fault detection method is about 40-times the corresponding kNN-based fault detection method.

## 5. Conclusions

The kNN-based fault detection methods, which can overcome the limitations of existing linear-based methods, are employed in more and more applications for reliable monitoring. To meet the requirements of real-time monitoring, a novel clustering-kNN rule is presented to enhance the efficiency of existing methods. In the process of off-line model construction, the LSC algorithm is adopted to divide the entire training sample set into several clusters. In the process of on-line fault detection, the nearest cluster of the test sample is firstly selected; then, all samples in it are utilized to conduct the kNN rule. With this method, the computational complexity of each fault detection method based on the proposed clustering-kNN rule is linear to the cluster size. The results of simulations and experiments indicate that the proposed clustering-kNN rule not only can reduce the time consumption of existing kNN-based methods, but also should be able to achieve similar performance on fault detection accuracy. We believe that it can provide a potential solution for reliable and real-time monitoring of gas sensor arrays.

In future work, we plan to verify and improve the robustness of the proposed fault detection methods under a complex noise environment. Moreover, we will focus on enhancing the reliability and maintainability of gas sensor arrays from the design stage; the robust fault detection filter (RFDF) design of gas sensor arrays with discrete time-varying delays [40] can be used to address this issue.

## Figures and Tables

**Table 1:** Fault detection results of the linear model.

Metrics	FD-kNN	C-FD-kNN	PC-kNN	C-PC-kNN	k-NND	C-k-NND	FS-kNN	C-FS-kNN
$T_{C}$ (s)	10.41	0.73	10.36	0.66	12.63	0.85	10.31	0.77
$T_{D}$ (ms)	5.20	0.29	5.20	0.30	6.34	0.34	5.19	0.22
DR (%)	85.9	85.5	69.7	69.9	82.6	82.5	84.8	84.2
FPR (%)	3.1	2.9	2.9	3.1	2.1	2.5	2.1	2.3

**Table 2:** Fault detection results of the non-linear model.

Metrics	FD-kNN	C-FD-kNN	PC-kNN	C-PC-kNN	k-NND	C-k-NND	FS-kNN	C-FS-kNN
$T_{C}$ (s)	2.57	0.31	2.56	0.33	3.09	0.37	2.57	0.33
$T_{D}$ (ms)	2.59	0.30	2.58	0.29	3.08	0.37	2.58	0.29
DR (%)	98.2	98.3	94.0	94.0	97.5	97.3	97.5	97.6
FPR (%)	0.8	1.3	2.1	2.3	0.9	1.4	1.6	2.0

**Table 3:** False positive detection rates.

Methods	PCA-Q	FD-kNN/C-FD-kNN	PC-kNN/C-PC-kNN	k-NND/C-k-NND	FS-kNN/C-FS-kNN
FPR (%)	1.6	1.8	2.9	3.5	3.9

**Table 4:** Model construction and detection time.

Metrics	FD-kNN	C-FD-kNN	PC-kNN	C-PC-kNN	k-NND	C-k-NND	FS-kNN	C-FS-kNN
$T_{C}$ (s)	160.82	5.13	157.20	4.47	200.15	5.70	157.13	4.48
$T_{D}$ (ms)	4.01	0.12	3.95	0.11	5.00	0.14	3.96	0.12
